# Patient Cost-Sharing and Utilization of Breast Cancer Diagnostic Imaging by Patients Undergoing Subsequent Testing After a Screening Mammogram

**DOI:** 10.1001/jamanetworkopen.2023.4893

**Published:** 2023-03-27

**Authors:** Danny R. Hughes, William Espinoza, Sarah Fein, Elizabeth Y. Rula, Geraldine McGinty

**Affiliations:** 1 College of Health Solutions, Arizona State University, Phoenix; 2 School of Economics, Georgia Institute of Technology, Atlanta; 3 Department of Radiology and Imaging Sciences, Emory University, Atlanta, Georgia; 4 now with Novant Health, Charlotte, North Carolina; 5 Georgia Institute of Technology, Atlanta; 6 now with Biofourmis, Inc., Boston, Massachusetts; 7 Harvey L. Neiman Health Policy Institute, Reston, Virginia; 8 Weill Cornell Medicine, New York City, New York

## Abstract

**Importance:**

Out-of-pocket costs (OOPCs) have been largely eliminated for screening mammography. However, patients still face OOPCs when undergoing subsequent diagnostic tests after the initial screening, which represents a potential barrier to those who require follow-up testing after initial testing.

**Objective:**

To examine the association between the degree of patient cost-sharing and the use of diagnostic breast cancer imaging after undergoing a screening mammogram.

**Design, Setting, and Participants:**

This retrospective cohort study used medical claims from Optum’s deidentified Clinformatics Data Mart Database, a commercial claims database derived from a database of administrative health claims for members of large commercial and Medicare Advantage health plans. The large commercially insured cohort included female patients aged 40 years or older with no prior history of breast cancer undergoing a screening mammogram examination. Data were collected from January 1, 2015, to December 31, 2017, and analysis was conducted from January 2021 to September 2022.

**Exposures:**

A k-means clustering machine learning algorithm was used to classify patient insurance plans by dominant cost-sharing mechanism. Plan types were then ranked by OOPCs.

**Main Outcomes and Measures:**

A multivariable 2-part hurdle regression model was used to examine the association between patient OOPCs and the number and type of diagnostic breast services undergone by patients observed to undergo subsequent testing.

**Results:**

In our sample, 230 845 women (220 023 [95.3%] aged 40 to 64 years; 16 810 [7.3%] Black, 16 398 [7.1%] Hispanic, and 164 702 [71.3%] White) underwent a screening mammogram in 2016. These patients were covered by 22 828 distinct insurance plans associated with 6 025 741 enrollees and 44 911 473 distinct medical claims. Plans dominated by coinsurance were found to have the lowest mean (SD) OOPCs ($945 [$1456]), followed by balanced plans ($1017 [$1386]), plans dominated by copays ($1020 [$1408]), and plans dominated by deductibles ($1186 [$1522]). Women underwent significantly fewer subsequent breast imaging procedures in dominantly copay (24 [95% CI, 11-37] procedures per 1000 women) and dominantly deductible (16 [95% CI, 5-28] procedures per 1000 women) plans compared with coinsurance plans. Patients from all plan types underwent fewer breast magnetic resonance imaging (MRI) scans than patients in the lowest OOPC plan (balanced, 5 [95% CI, 2-12] MRIs per 1000 women; copay, 6 [95% CI, 3-6] MRI per 100 women; deductible, 6 [95% CI, 3-9] MRIs per 1000 women.

**Conclusions and Relevance:**

Despite policies designed to remove financial barriers to access for breast cancer screening, significant financial barriers remain for women at risk of breast cancer.

## Key Points

**Question:**

Do individuals with higher cost-sharing undergo fewer subsequent breast diagnostic tests after a screening mammogram compared with those with lower cost-sharing?

**Findings:**

In this cohort study of 230 845 commercially insured women undergoing a mammogram in 2016, patients with plans with higher out-of-pocket costs underwent significantly fewer subsequent diagnostic breast procedures than patients enrolled in plans with lower out-of-pocket costs.

**Meaning:**

The findings of this study suggest that out-of-pocket costs continue to be a barrier for early diagnosis of breast cancer despite the removal of cost-sharing from initial breast cancer screening examinations.

## Teaser

This cohort study examines the association between the degree of patient cost-sharing and the use of diagnostic breast cancer imaging after undergoing a screening mammogram.

## Introduction

Out-of-pocket costs (OOPCs) are used by insurers to mitigate moral hazard by patients, ie, reduce the likelihood patients will undertake riskier behaviors or excessively seek care after acquiring health insurance.^1^ High OOPCs have been shown to reduce health care utilization, but they can also induce patients to either defer or underutilize needed care, such as recommended preventive services.^2,3,4,5^ Because of these concerns, provisions within the Patient Protection and Affordable Care Act (ACA) and Protecting Access to Lifesaving Screenings (PALS) Act removed OOPCs for screening mammography for women aged 40 years and older.^6,7,8^

Despite the elimination of OOPCs for screening mammography, patients still face OOPCs if they undergo further diagnostic tests—costs that have been increasing over time.^9^ Women in high-deductible plans, which historically have higher OOPCs than other plan types,^1,10,11^ have been found to experience delays in follow-on care, such as diagnostic breast imaging and biopsies.^12,13,14^ However, little is known about the association of high-deductible plans (or other kinds of commercial insurance plans) with patient decisions to undergo recommended follow-on diagnostic testing. Although most of this subsequent care ultimately finds an initial abnormal screening mammogram to be a false positive,^15^ with additional diagnostic services recommended in more than 10% of screening mammograms,^9,16^ if patient concerns regarding OOPCs are deterring women from undergoing recommended follow-on testing, this has the potential to adversely affect outcomes for a substantial number of those who require follow-up testing after the initial test.

This study applies a novel machine learning approach to a retrospective national commercial claims data set to infer the cost-sharing mechanism for patients’ OOPCs for their health plans, as would be found on their health insurance cards. It should be noted that a patient’s actual OOPCs can vary from those expected (ie, the OOPC listed on their health insurance cards) because of a variety of factors that include covered health care networks and the payer’s determination of coverage for specific medical procedures, among others. However, we assume patients consider their expected OOPCs when deciding to seek care, given that their actual OOPCs are typically unknown until reaching the point of service—and often not until several weeks afterwards. We use our inferred cost-sharing mechanism to investigate the association between expected OOPCs and their use of follow-on diagnostic breast services for patients who undergo subsequent testing after a screening mammogram.

## Methods

To examine the association between expected patient cost-sharing and subsequent breast cancer diagnostic tests, we extracted data from Optum’s deidentified Clinformatics Data Mart (CDM), which contains health insurance claims for members of large commercial and Medicare Advantage health plans. The CDM is an administrative database, covering a geographically diverse population, including all 50 states and the District of Columbia. The Georgia Institute of Technology institutional review board deemed this retrospective study of deidentified administrative claims data as not constituting human participant research and thus waived review and the requirement for informed consent. This study follows the Strengthening the Reporting of Observational Studies in Epidemiology (STROBE) reporting guideline.

### Study Cohort

Our study cohort was restricted to female patients continuously enrolled in the same insurance plan during the study period (2015-2017), who were 40 years or older at the beginning of the study period, alive at the end of the study period, and had a screening mammogram (*Common Procedural Terminology *[*CPT*] codes G0202, 77057, and 77063) in 2016, which served as the index event for an episode of care. Episodes were terminated either (1) 365 days after the index screening mammogram or (2) the earliest date a patient has a claim reporting a diagnosis of breast cancer within 365 days of the index event. We removed any patients who reported a diagnosis of breast cancer (*International Classification of Diseases, Ninth Revision *[*ICD-9*] codes, 174.x; *International Statistical Classification of Diseases and Related Health Problems, Tenth Revision *[*ICD-10*] codes, C50.x) in the 12 months preceding the 2016 screening mammogram. For each patient in the resulting study cohort, we identified claims related to diagnostic breast imaging occurring within the episode of care. The breast imaging claims were grouped into 3 broad service categories: diagnostic mammography (*CPT* codes G0204 and G0206), breast ultrasonography (*CPT *codes 76641 and 76642), and breast magnetic resonance imaging (MRI; *CPT *code 77059). The total number of subsequent breast imaging services was tabulated for each service category. We separately tabulated the total number of breast biopsies (*CPT* codes in eTable 1 in Supplement 1) in each episode given their importance for establishing a definitive diagnosis and which could occur at any point after the screening mammogram or subsequent diagnostic imaging.

We defined a patient undergoing subsequent diagnostic testing as any patient who underwent a breast imaging service or breast biopsy within the episode of care. We then characterized the study cohort, stratified by whether they underwent subsequent testing, by race and ethnicity (Asian, Black, Hispanic, White, and unknown) and age (40-64, 65-74, 75-84, and ≥85 years). These covariates were included to examine the distribution among plan types. We also calculated and reported prospective Charlson Comorbidity Scores (0, 1, 2, and ≥3) using patients’ medical claims from the preceding year to account for patient health status.^17^ Patient demographics were reported in the CDM as defined by the patient.

### Insurance Mechanism Design

Like most administrative claims data sets, the CDM contains the amount of patient cost-sharing associated with individual claims but does not contain the individual patient’s overall insurance mechanism design, ie, the specific amounts or percentages that patients must pay in copays, coinsurance, and deductibles at the point of service and reported on a patient’s insurance card. However, because the CDM contains a 100% sample of all enrollees’ claims within each insurance plan, as well as a unique identifier for plans, we can infer mechanism design by examining patient cost-sharing for all enrollees with a matching insurance group number as each patient within our study cohort. We are then able to use a k-means clustering machine learning algorithm to classify each specific plan by the dominant category of out-of-pocket spending, ie, dominantly characterized by either copays, coinsurance, or deductibles, or as being balanced among the 3 cost-sharing categories. These 4 plan types can then be ranked by the relative degree of OOPCs their enrollees face. This process is described as follows.

For each patient in the study cohort, we examined all claims associated with all enrollees in that patient’s specific plan to calculate the mode copay, mode coinsurance percentage (ie, coinsurance amount / total reimbursement), and maximum deductible paid across all enrollees in the plan. Total reimbursement is measured by the reported standard average cost reported on the claim. Each plan’s mechanism design is then characterized by the triplet of parameters: mode copay, mode coinsurance percentage, maximum deductible.

We then used a k-means clustering algorithm to classify plans into 4 plan types characterized by whether the plan’s mechanism design is dominated by patient (1) copays, (2) coinsurance, or (3) a deductible as well as plans that are (4) relatively balanced across the 3 cost-share components. The k-means clustering approach is a popular iterative descent unsupervised learning algorithm that assigns data points to discrete clusters where the dissimilarity between data points within a cluster is minimized.^18^ To ensure that cluster assignment would not be determined by a single dominant parameter, eg, deductibles, we scaled each triplet parameter to a value between 0 and 1 using min-max scaling: (Maximum Value of All Plans − Plan Value) / (Maximum Value of All Plans − Minimum Value of All Plans).

The k-means function initializes 3 random cluster centers and 1 deterministic cluster at (0, 0, 0). It then assigns all plans to the nearest cluster center using a k-dimensional tree that defines closeness as the Euclidean distance from 2 points. After all plans have been assigned, the cluster centers are recalculated as the average triplet point of all insurance groups assigned to the cluster. Plans are then reassigned to clusters using these new cluster centers. This process is repeated until no cluster assignments change and all plans are classified as balanced cost-sharing, dominated by copays, dominated by coinsurance, or dominated by deductibles. Finally, we ranked each plan type by their relative degree of patient cost-sharing by first calculating the mean of the total annual OOPC for each enrollee in each plan and then calculating the average of each of these resulting plan averages for the plans in each of the 4 plan types generated by the k*-*means clustering process.

### Statistical Analysis

Because most women undergoing screening mammograms do not undergo further testing until their next screening mammogram (eg, the National Mammography Database has a mean recall rate of 10.6%),^19^ we estimated the association of patient cost-sharing with the utilization of additional diagnostic breast imaging and breast biopsy with a 2-part hurdle regression model.^20^ The first part uses a logistic regression to estimate the likelihood that a patient underwent any subsequent diagnostic breast imaging or biopsy. The second part uses a zero-truncated negative binomial regression model to estimate the association between the number of subsequent breast diagnostic services a patient undergoes among those undergoing additional testing and the relative degree of cost-sharing of the patient’s insurance plan type (eg, balanced cost-sharing, dominated by copays, dominated by coinsurance, or dominated by deductibles) relative to the plan type with the lowest OOPCs. We estimate this model separately for different outcomes, specifically, the number of (1) all subsequent breast imaging, (2) diagnostic breast imaging, (3) breast ultrasonography, (4) breast MRI, and (5) breast biopsy. Both regression stages are adjusted for the following potential confounders: race (Asian, Black, Hispanic, and unknown, with White as the reference category), age (65-74 and ≥75 years, with 40-64 years as the reference category), prospective CCI score (1, 2, and ≥3, with 0 as the reference category), and patient’s state of residence. Estimates of the marginal effect for the change in the number of services associated with cost-sharing are extracted using the delta method with Duan smearing.^21^

Data management was performed with SAS version 9.4 (SAS Institute), the k-means clustering analysis was conducted with Python version 3.7 (Python Software Foundation), and all statistical analyses were performed with Stata version 16 (StataCorp). Two-sided tests with α = .05 were used for testing statistical significance.

## Results

The CDM contained 19 117 756 enrollees in 2016. There were 9 724 515 (50.9%) female enrollees, of whom 5 502 691 were aged 40 years or older during the year (eTable 2 in Supplement 1). The study cohort contained 230 845 women who had a screening mammogram in 2016 and met the study inclusion criteria. Nearly all participants were aged 40 to 64 years (220 023 [95.3%]); 8279 (3.6%) were Asian, 16 810 (7.3%) were Black, 16 398 (7.1%) were Hispanic, and 164 702 (71.3%) were White. These women were covered by 22 828 distinct insurance plans with a total of 6 025 741 enrollees and 44 911 473 distinct medical claims. The distribution of women in the study cohort by race, age, and prospective CCI is reported in Table 1 as well as the mean total medical OOPCs and mean total OOPCs on breast imaging services. Of the 230 845 women undergoing a screening mammogram, 25 073 (10.9%) had additional diagnostic imaging. Patient demographic characteristics were similar between women in this group and those with no additional testing. Women undergoing additional imaging paid a mean (SD) of $75.24 ($153.82) in OOPCs for breast imaging and $1401.54 ($1611.81) in annual OOPCs for all medical services vs $1.13 ($12.21) for breast imaging and $1055.42 ($1449.16) for all medical services for women only undergoing a screening mammogram.

**Table 1.** Demographic Characteristics and Out-of-Pocket Costs of Patients Undergoing a Screening Mammogram in 2016

Variable	Patients, No. (%)		
	Full sample (N = 230 845)	Screening only (n = 205 772 [89.1%])	Subsequent imaging (n = 25 073 [10.9%])
Race and ethnicity			
Asian	8279 (3.6)	7361 (3.6)	918 (3.7)
Black	16 810 (7.3)	14 992 (7.3)	1818 (7.3)
Hispanic	16 398 (7.1)	14 399 (7.0)	1999 (8.0)
White	164 702 (71.3)	147 214 (71.5)	17 488 (69.7)
Unknown	24 656 (10.7)	21 806 (10.6)	2850 (11.4)
Age, y			
40-64	220 023 (95.3)	195 904 (95.2)	24 119 (96.2)
65-74	10 348 (4.5)	9435 (4.6)	913 (3.6)
75-84	458 (0.2)	419 (0.2)	39 (0.2)
≥85	16 (<0.01)	14 (<0.01)	2 (<0.01)
CCI score			
0	201 648 (87.4)	179 584 (87.3)	22 064 (88.0)
1	24 064 (10.4)	21 584 (10.5)	2470 (9.9)
2	4047 (1.8)	3621 (1.8)	426 (1.7)
≥3	1086 (0.5)	973 (0.5)	113 (0.5)
Out-of-pocket costs, mean (SD), $			
All medical	1093.01 (1471.65)	1055.42 (1449.16)	1401.54 (1611.81)
Breast imaging	9.18 (56.87)	1.13 (12.21)	75.24 (153.82)

Abbreviation: CCI, Charlson Comorbidity Index.

The k-means clustering algorithm classified the 22 828 plans as follows: 4736 (20.7%) were balanced plans, 13 310 (58.3%) were dominated by copays, 1881 (8.2%) were dominated by coinsurance, and 2901 (12.7%) were dominated by deductibles (Table 2). However, patients in the study cohort were not similarly distributed across plan types; in particular, a much larger proportion (116 230 [50.3%]) were in dominantly deductible plans and a much lower proportion in dominantly coinsurance plans (30 911 [13.4%]) (Table 2). The distribution of study cohort patients across plan types was similar between patients who only underwent screening and those who underwent subsequent diagnostic testing. The median (IQR) number of patients across all plans was 1 (1-3). Cost-sharing components by plan type are reported in eTable 3 in Supplement 1. Ranking plans from lowest to highest mean annual OOPCs (Table 3) for all medical services found that plans dominated by coinsurance had the lowest mean (SD) OOPCs ($945.36 [$1456.36]), followed by balanced plans ($1017.07 [$1385.65]), plans dominated by copays ($1020.31 [$1408.27]), and plans dominated by deductibles ($1186.02 [$1522.39]).

**Table 2.** Distribution of Plans and Patients Across Plan Types

Plan type	Plans in study sample, No. (%) (N = 22 828)	Patients, No. (%)			Study patients per plan, median (IQR)
		Full sample (N = 230 845)	Screening only (n = 205 772)	Subsequent testing (n = 25 073)	
Blended	4736 (20.7%)	49 683 (21.5%)	44 554 (21.7%)	5129 (20.5%)	2 (1-4)
Copay	13 310 (58.3%)	34 021 (14.7%)	30 289 (14.7%)	3732 (14.9%)	1 (1-1)
Coinsurance	1881 (8.2%)	30 911 (13.4%)	27 341 (13.3%)	3570 (14.2%)	2 (1-6)
Deductible	2901 (12.7%)	116 230 (50.3%)	103 588 (50.3%)	12 642 (50.4%)	2 (1-8)

**Table 3.** Distribution of Out-of-Pocket Costs by Plan Type

Plan type	All medical		Breast imaging services		Breast biopsy	
	Mean	Median (IQR)	Mean	Median (IQR)	Mean	Median (IQR)
Balanced	1017.07 (1385.65)	510.92 (150.00-1394.52)	7.55 (51.49)	0 (0-0)	20.58 (162.56)	0 (0-0)
Copay	1020.31 (1408.27)	490.87 (150.00-1409.61)	7.71 (54.78)	0 (0-0)	24.84 (191.09)	0 (0-0)
Coinsurance	945.36 (1456.36)	433.41 (137.50-1264.50)	6.37 (51.20)	0 (0-0)	19.70 (169.15)	0 (0-0)
Deductible	1186.02 (1522.39)	629.43 (195.00-1640.73)	11.05 (60.89)	0 (0-0)	27.62 (192.98)	0 (0-0)
Total	1093.01 (1471.65)	553.1 (167.36-1501.99)	9.18 (56.87)	0 (0-0)	15.46 (165.26)	0 (0-0)

Data on OOPCs and breast diagnostic service utilization, stratified by patients who only underwent a screening mammogram and those that underwent subsequent diagnostic testing, are reported in Table 4 in ranked order from plans with lowest overall OOPCs (coinsurance) to highest overall OOPCs (deductible). The overall unadjusted rate of subsequent testing was observed to range between 5209 patients (10.5%) in balanced plans to 3780 patients (12.1%) in dominantly copay plans. For patients undergoing additional diagnostic testing, the mean (SD) number of additional breast imaging services ranged from 1.91 (1.02) in dominantly deductible plans to 1.96 (1.07) in dominantly coinsurance plans, with most patients who underwent further tests across all plan types having at least 1 subsequent diagnostic mammogram and 1 breast ultrasound. The use of breast MRI decreased 23.8% between patients undergoing subsequent testing in plans with the lowest cost-sharing (dominantly coinsurance, 320 patients [8.5%]) and those in plans with the highest cost sharing (dominantly deductible, 828 patients [6.4%]). The use of breast biopsy was very similar across all plan types: 1.9% in dominantly coinsurance and balanced plans, 2.1% in dominantly copay plans, and 2.0% in dominantly deductible plans.

**Table 4.** Number and Type of Downstream Breast Services Performed By Plan Type, Ordered by Plans With Lowest to Highest Overall Medical Out-of-Pocket Costs for the Full Sample

Variable	Coinsurance		Balanced		Copay		Deductible	
	Screening only	Subsequent testing	Screening only	Subsequent testing	Screening only	Subsequent testing	Screening only	Subsequent testing
Patients, No. (%)	27 341 (87.9)	3780 (12.1)	44 554 (89.5)	5209 (10.5)	30 289 (88.9)	3780 (11.1)	103 588 (89.0)	12 848 (11.0)
Out-of-pocket costs, mean (SD), $								
All medical	917.66 (1460.55)	1157.48 (1406.10)	981.90 (1353.78)	1322.63 (1604.67)	986.87 (1394.07)	1291.65 (1491.33)	1143.44 (1495.24)	1534.92 (1689.03)
Breast imaging	1.55 (18.15)	43.32 (136.50)	0.95 (9.35)	64.90 (145.79)	1.00 (11.56)	62.15 (151.50)	1.13 (11.51)	92.31 (159.99)
Service performed								
Diagnostic mammogram	0	2695 (71.3)	0	3871 (74.3)	0	2805 (74.2)	0	9325 (72.6)
Breast ultrasound	0	2563 (67.8)	0	3488 (67.0)	0	2522 (66.7)	0	8648 (67.3)
Breast MRI	0	320 (8.5)	0	344 (6.6)	0	259 (6.9)	0	828 (6.4)
Breast biopsy	0	72 (1.9)	0	99 (1.9)	0	709 (2.1)	0	257 (2.0)
No. of downstream services, mean (SD)								
Any breast images	0	1.96 (1.07)	0	1.92 (1.05)	0	1.93 (1.06)	0	1.91 (1.02)
Diagnostic mammogram	0	0.82 (0.64)	0	0.87 (0.64)	0	0.88 (0.66)	0	0.84 (0.63)
Breast ultrasound	0	0.92 (0.81)	0	0.88 (0.79)	0	0.87 (0.78)	0	0.88 (0.78)
Breast MRI	0	0.09 (0.32)	0	0.07 (0.29)	0	0.08 (0.29)	0	0.07 (0.29)
Breast biopsy	0	0.05 (0.37)	0	0.04 (0.36)	0	0.05 (0.41)	0	0.04 (0.37)

Abbreviation: MRI, magnetic resonance imaging.

Estimates of the association between plan type and utilization of breast diagnostic services for women undergoing subsequent diagnostic testing relative to the plan type with lowest OOPCs (ie, dominantly coinsurance plans) are reported in Table 5. Patients in dominantly copay plans underwent on average 24 fewer subsequent breast imaging procedures per 1000 patients (−0.024; 95% CI, −0.037 to −0.011) than those in dominantly coinsurance plans; women in dominantly deductible plans underwent 16 fewer per 1000 patients (−0.016; 95% CI, −0.028 to −0.005). Similar statistically significant results were found for subsequent diagnostic mammogram services (copay: −0.011; 95% CI, −0.020 to −0.002; deductible: −0.008; 95% CI, −0.016 to −0.000). Among plan types, only patients with dominantly copay plans experienced a statistically different number of breast ultrasonographic scans than the lowest OOPC plan, with 9 fewer per 1000 patients (−0.009; 95% CI, −0.018 to −0.001). Patients from all plan types were estimated to undergo significantly fewer breast MRIs than patients in the lowest OOPC plan (balanced: −0.005; 95% CI, −0.012 to −0.002; copay: −0.006; 95% CI, −0.006 to −0.003; deductible: −0.006; 95% CI, −0.009 to −0.003). There was no observed statistically significant difference in breast biopsy utilization between plan types.

**Table 5.** Estimate of the Marginal Effect of Plan Type on the Utilization of Diagnostic Breast Services For Patients Undergoing Subsequent Testing Relative to Plan Type With Lowest Out-of-Pocket Cost (ie, Dominantly Coinsurance)^a^

Plan type and outcomes	Estimate (95% CI)	*P* value
Any subsequent breast image		
Balanced	−0.007 (−0.02 to 0.01)	.35
Dominantly copay	−0.024 (−0.04 to −0.01)	<.001
Dominantly deductible	−0.016 (−0.03 to −0.01)	.01
Diagnostic mammogram		
Balanced	−0.001 (−0.01 to 0.01)	.75
Dominantly copay	−0.011 (−0.02 to −0.00)	.02
Dominantly deductible	−0.008 (−0.02 to −0.00)	.04
Breast ultrasound		
Balanced	−0.000 (−0.01 to 0.01)	.94
Dominantly copay	−0.009 (−0.02 to −0.00)	.03
Dominantly deductible	−0.005 (−0.01 to 0.00)	.23
Breast MRI		
Balanced	−0.005 (−0.01 to −0.00)	<.001
Dominantly copay	−0.006 (−0.01 to −0.00)	<.001
Dominantly deductible	−0.006 (−0.01 to −0.00)	<.001
Breast biopsy		
Balanced	−0.009 (−0.02 to −0.00)	.05
Dominantly copay	−0.000 (−0.011 to −0.010)	.94
Dominantly deductible	−0.005 (−0.01 to 0.00)	.29

Abbreviation: MRI, magnetic resonance imaging.

^a^ Estimates adjusted for patient age, race and ethnicity, prospective Charlson Comorbidity Index, and state of domicile.

## Discussion

Using a large administrative claims database, we were able to use a novel machine learning approach to infer patients’ health insurance plan’s cost-sharing mechanisms and then classify and rank their plan types by their degree of OOPCs. It is well known that deductible-oriented (commonly known as high-deductible) plans have higher OOPCs per medical visit than non–high-deductible plans.^11,22^ We found that there is a distinct ordering between patient cost-sharing and other plan types, with lower patient cost-sharing associated with dominantly coinsurance plans, followed by dominantly copay plans and then dominantly deductible plans. This may be important information for consumers when assessing the trade-off between different plans’ cost-sharing mechanisms and premiums when making insurance decisions.

Previous studies have shown that patients in high-deductible plans reduce their use of screening mammography—even though this service requires no patient cost-sharing—and experience delays in undergoing subsequent diagnostic breast imaging,^12,13,14^ indicating patients consider the additional costs of potential follow-up testing when deciding to undergo an initial screen or subsequent procedure.^9^ However, little is known about the association between patient cost-sharing and utilization for patients with different cost-sharing mechanisms in non–high deductible plans. Although patients were not observed to reduce their utilization of breast biopsy, we found that patients in plans with higher degrees of cost-sharing had lower utilization of subsequent diagnostic imaging and that this was more pronounced for the most expensive service studied (ie, breast MRI). Considering the risk posed by an unconfirmed positive mammogram result, this is a startling finding that questions the efficacy of legislation such as PALS and ACA, which eliminated cost-sharing from many preventive services, such as screening mammograms, precisely to remove financial barriers that inhibit patients from receiving these important life-saving services. Additionally, because most abnormal results from screening mammograms are ultimately costly false positives,^15,23^ strictly adhering to physician recommendations financially penalizes patients with abnormal screening results.

One possible solution could be to expand the elimination of patient cost-sharing to subsequent testing when recommended after the initial screening results, as was recently implemented by the Biden administration for subsequent procedures associated with abnormal initial colorectal cancer screening results.^24^ An alternative solution may be bundling screening mammograms with subsequent diagnostic testing, as others have proposed and which has been shown to only modestly increase the overall cost to payers while simultaneously removing financial barriers to patients and incentivizing clinicians to avoid excessive utilization.^25,26,27^

### Limitations

This study has limitations. The CDM contains data from commercial and Medicare Advantage plans, which may not be generalizable to other commercial and traditional Medicare enrollees, although this is mitigated by the large number of distinct plans (22 828) in our study sample. We also cannot directly observe physician recommendations for subsequent diagnostic testing. Because any patients not following up on a recommended diagnostic test would be included in the initial mammogram–only sample in the analysis, our findings are downward biased and represent an underestimate of the underlying association. However, our rate of subsequent diagnostic imaging after undergoing a screening mammogram (10.9%) is consistent with other studies using different data sets,^16,19,25^ minimizing this potential bias. Moreover, with large samples of patients in each of the 4 plan types (range, 30 911-116 230), the unobserved distributions of recommended subsequent services and patient characteristics in each plan type should converge across plan types, allowing observed variation in services across plan types to be attributed to the variation in cost-sharing. We saw no evidence of this selection in the distribution of enrollees across plan types by whether women underwent subsequent testing (Table 2). A portion of the subsequent observed imaging may reflect supplemental screening for women with very high risk or dense breasts. We discuss this limitation further in the eAppendix in Supplement 1.

## Conclusions

Despite policies designed to remove financial barriers to access for breast cancer screening, significant financial barriers remain for women at risk of an abnormal screening test. Further research should examine whether patient cost-sharing is associated with subsequent testing in other cancer screening efforts, as it is likely the financial costs of subsequent testing informs patient adherence to physician recommendations in other clinical contexts. Additional policy changes, such as removing cost-sharing for subsequent tests after abnormal screening results or bundling all breast cancer diagnostic testing into a single reimbursement, may provide avenues to mitigate these financial barriers to care.

## Notes

Supplement 1. **eAppendix. **Expansion on Methods and Limitations **eTable 1.** Breast Biopsy CPT Codes **eTable 2.** Patient Demographics from 2016 CDM Cohort **eTable 3.** Cost-Sharing Components by Plan Type

Supplement 2.
Data Sharing Statement
