# Supplementary material for: Patient Cost-Sharing and Utilization of Breast Cancer Diagnostic Imaging by Patients Undergoing Subsequent Testing After a Screening Mammogram
Source: JAMA Netw Open. 2023 Mar 27;6(3):e234893. doi: 10.1001/jamanetworkopen.2023.4893 (PMC10043745; doi:10.1001/jamanetworkopen.2023.4893)
Supplement: Supplement 1. — eAppendix. Expansion on Methods and Limitations eTable 1. Breast Biopsy CPT Codes eTable 2. Patient Demographics from 2016 CDM Cohort eTable 3. Cost-Sharing Components by Plan Type [file jamanetwopen-e234893-s001.pdf]

Supplemental Online Content

Hughes DR, Espinoza W, Fein S, Rula EY, McGinty G. Patient cost-sharing and utilization of breast cancer diagnostic imaging by patients undergoing subsequent testing after a screening mammogram. *JAMA Netw Open.* 2023;6(3):e234893. doi:10.1001/jamanetworkopen.2023.4893

eAppendix. Expansion on Methods and Limitations

eTable 1. Breast Biopsy CPT Codes

eTable 2. Patient Demographics from 2016 CDM Cohort

eTable 3. Cost-Sharing Components by Plan Type

This supplemental material has been provided by the authors to give readers additional information about their work.

© 2023 Hughes DR et al. *JAMA Network Open.*

eAppendix. Expansion on Methods and Limitations

Identifying Effect of Patient Cost-Sharing on Utilization

Studies examining the effects of payments and healthcare utilization are usually plagued by endogeneity, i.e. consumers inclined to use fewer health care resources will select into less generous plans making it difficult to assess whether lower utilization is related to the higher out-of-pocket costs or simply consumer preferences to useless medical care. Another confounder for studies examining adherence to physician recommendations is that claims data does not provide information on the actual physician recommendation. Researchers only observe the follow-up care for patients that did adhere.

However, we are able to address these concerns for subsequent breast cancer testing if the following assumptions are met:

1. Patients can be grouped into different samples that vary only by the degree of cost-sharing;
2. Selection of patients into these groups are not correlated with the risk of acquiring the clinical condition (e.g. breast cancer);
3. Physicians refer for additional testing based on an abnormal test result without regard for patient cost-sharing; and
4. The sample size of each of the groups are sufficiently large to appeal to asymptotic statistical properties;

then the distribution of women actually recommended for subsequent testing, should be statistically equivalent in each group. Therefore, any variation in the expected value of tests (overall or by test type) between groups can be attributed to the degree of cost-sharing between groups – even though we cannot directly observe the physician recommendations.

By using the *k*-means cluster algorithm to group patients into plans dominated by one specific type of cost-sharing (copay, coinsurance, deductible, and balanced), assumptions #2 and #4 allow us to directly test the distributions of characteristics between groups. We find the observables (e.g. age, CCI, distribution between screening only and undergoing further tests, etc.) to be similar (**Tables 1 & 2**) and argue that these assumptions are reasonable. Under this approach we can not directly test patient OOP cost and their utilization without incurring problems of endogeneity – but as long as the groups consistently vary in their degree of cost-sharing, we can interpret the effect of being in a specific group on utilization as the effect of OOP cost on utilization (under the assumptions above).

Inferring Mechanism Design

The primary problem faced in inferring mechanism design and grouping plans into informative groups in the that virtually all plans incorporate some facet of all three cost-sharing components. We should note that there is no need to define specific plan types at all as long as the 4

© 2023 Hughes DR et al. *JAMA Network Open.*

assumptions above are met. One could simply increase the number of clusters employed by the *k*-means clustering algorithm to the largest number available such that the number of observations in the smallest cluster was large enough to ensure there are balanced covariates across all clusters and sufficiently powered to appeal to asymptotic statistical properties. We choose to use 4 clusters defined by plan type in this study for two reasons:

1. Examining by plan types is more intuitive than a large number of undefined "clusters"
2. While many studies have examined patient behavior between enrollees of high-deductible and non-high deductible plans, to our knowledge, no study has examined what occurs between different non-high deductible insurance plan types.

Thus, although virtually all insurance plans incorporate all three forms of cost-sharing, we choose to group plans into intuitive, interpretable groups: those where dominant (i.e. majority of OOP expenditures) are either co-pays, co-insurance payments, or deductibles. Plans where there is no clear weight towards one of these are allocated by the clustering process to what we refer to as a "balanced" plan type. The *k*-means clustering process allocates plans into the other three groups by the relative weighting of the three facets towards one of these groups.

Analysis

Once groups have been established, we estimate the effect of cost-sharing on utilization by regressing plan types (characterized by different degree of OOP costs) on utilization using a two-part "hurdle" model. The first stage is a regression that examines the association of plan type on whether a patient is observed to undergo a subsequent breast diagnostic imaging or biopsy procedure. This second stage runs a regression on the sub-sample of patients that underwent subsequent testing to examine the association between plan type and intensity (i.e. the number) of subsequent procedures. These two separate effects are then combined into a single interpretable marginal effect that incorporates both the external margin (i.e., likelihood one undergoes a subsequent test) and the internal margin (i.e., conditional on undergoing subsequent testing, how many additional tests did the patient undergo). These are the results reported in **Table 5**.

Limitations

Supplemental screening associated with i) very high-risk women not removed by our exclusion of patients reporting a breast cancer diagnosis in the previous year or ii) women with dense breasts, would be represented in the sample and grouped with other subsequent breast imaging. The effect of this is unclear. As discussed in the Methods section of the manuscript and this supplemental appendix, we work under the assumption that physicians' decisions to recommend subsequent tests are not correlated with the out-of-pocket costs associated with their patient's plans. We also provide evidence that the distribution of patient characteristics is very similar between plan types as this assumption implies. By this reasoning, we do not believe that the share of women receiving recommendations for supplemental screening for these two reasons – rather than supplemental diagnostic imaging – would differ between the plan types.

© 2023 Hughes DR et al. *JAMA Network Open.*

If we were to believe that women with dense breasts or of very high-risk select into different plan types *a prior*, this would no longer be valid. Again, we see no evidence of self-selection along observable characteristics into different plan types in our study cohort. However, this does present a different question: Even if a consistent share of women receiving supplemental testing is for *supplemental screening* rather than *diagnostic testing*, how does the inclusion of these tests bias the analysis?

To our knowledge, patients are required to pay out-of-pocket costs for these supplemental tests regardless of rationale, implying the estimates still reflect the decrease in *observed subsequent testing* – though the source and reasoning of the recommendation to undergo a subsequent test may differ. This does imply that the nominal estimates (e.g. patients in dominantly copay plans underwent on average 24 fewer subsequent breast imaging procedures per 1000 patients than those in dominantly co-insurance plans) would over-estimate the effect of out-of-pocket costs on *subsequent diagnostic testing* as a portion of this reduction would be attributed to fewer *supplemental screening tests*. We leave the importance of that distinction to the interpretation of the readers. We feel this isn't material, given that the statistical correlation of the reduction between plan types would not be biased by this effect if we can appeal to the assumption that the share of women receiving recommendations for supplemental screening are not different between insurance plan types because our statistical estimates are invariant to monotonic transformations of the outcome variables. Unfortunately, there is no method to identify and decompose the reduction in *subsequent testing* into separate estimates for *supplemental screening* and *supplemental diagnostic testing.*

It has been suggested by a reviewer that the relatively higher rates of subsequent MRI imaging – which does not have a substantial role in the evaluation of abnormal mammograms and is more frequently used for *supplemental screening* – among patients in plans with lower cost sharing may reflect overuse of these services when jointly interpreted with the lack of a statistical correlation between plan type and the use of subsequent breast biopsy. It is entirely possible that subsequent MRI imaging is over-used (or under-used), but we do not believe that the results of this analysis provide evidence in either direction unless we believe that physicians are identifying patients with relatively lower and higher out-of-pocket costs and making differential recommendations for subsequent MRI to these groups for the reasons underpinning our study design. We conjecture that patients may interpret a recommended invasive procedure, such as breast biopsy, with more seriousness than a recommendation to "take another look" with subsequent imaging – given that the patient could have been recommended to move directly to a breast biopsy after screening instead. Of course, this conjecture is not supported by our observed evidence – which is beyond the scope of this investigation – and we leave interpretation of these results and possible counter-factual theories to readers and future research.

© 2023 Hughes DR et al. *JAMA Network Open.*

eTable 1. Breast Biopsy *CPT* Codes

19000, 19001, 19100, 19102, 19081-19086, 19120, 19125, 19126, 19101, 0HBT0ZZ, 0HBT3ZZ 0HBT0ZX, 0HBT3ZX, 0HBU0ZZ, 0HBU3ZZ, 0HBU0ZX, 0HBU3ZX 0HBV0ZZ, 0HBV3ZZ, 0HBV0ZX, 0HBV3ZX, 0HBW0ZZ, 0HBW3ZZ, 0HBWXZZ, 0HBW0ZX, 0HBW3ZX, 0HBWXZX 0HBX0ZZ, 0HBX3ZZ, 0HBXXZZ, 0HBX0ZX, 0HBX3ZX, 0HBXXZX

© 2023 Hughes DR et al. *JAMA Network Open.*

eTable 2. Patient Demographics from 2016 CDM Cohort

			Enrollee Age			
**Sex**	**Age**	**N**	**Mean**	**Std Dev**	**Min**	**Max**
**All**	0-17	3,076,380	8.84	5.16	0	17
	18-39	5,607,309	29.02	6.07	18	39
	40-64	6,193,036	51.79	7.04	40	64
	65+	4,241,031	74.36	7.11	65	91
	All	19,117,756	43.21	23.04	0	89
**Female**	0-17	1,503,836	8.86	5.16	0	17
	18-39	2,717,988	29.00	6.07	18	39
	40-64	3,091,534	51.92	7.04	40	64
	65+	2,411,157	74.69	7.28	0	89
	All	9,724,515	44.50	23.46	0	89
**Male**	0-17	1,572,544	8.82	5.16	0	17
	18-39	2,889,321	29.03	6.07	18	39
	40-64	3,101,502	51.67	7.04	40	64
	65+	1,829,874	73.91	6.85	65	89
	All	9,393,241	41.87	22.51	0	89

© 2023 Hughes DR et al. *JAMA Network Open.*

eTable 3. Cost-Sharing Components by Plan Type

Plan Type	# Plans	Copay		Co-Insurance		Deductible	
		Mean	Mode	Mean	Mode	Mean	Max
Blended	4736	32.79	25.00	7%	10%	1803.20	1000.00
Copay	13310	30.09	25.00	21%	15%	2898.48	1000.00
Co-insurance	1881	30.51	25.00	52%	60%	3470.27	1500.00
Deductible	2901	33.36	30.00	10%	12%	6858.28	5000.00

© 2023 Hughes DR et al. *JAMA Network Open.*
