# Supplementary material for: Patient Cost-Sharing and Utilization of Breast Cancer Diagnostic Imaging by Patients Undergoing Subsequent Testing After a Screening Mammogram
Source: JAMA Netw Open. 2023 Mar 27;6(3):e234893. doi: 10.1001/jamanetworkopen.2023.4893 (PMC10043745; doi:10.1001/jamanetworkopen.2023.4893)
Supplement: Supplement 2. — Data Sharing Statement [file jamanetwopen-e234893-s002.pdf]

Data Sharing Statement

Hughes. Patient Cost-Sharing and Utilization of Breast Cancer Diagnostic Imaging by Patients Undergoing Subsequent Testing After a Screening Mammogram. *JAMA Netw Open*. Published March 27, 2023. doi:10.1001/jamanetworkopen.2023.4893

Data
Data available: No

Additional Information
Explanation for why data not available: Our data use agreement with Optum does not permit sharing of this 8GB database
